# Measuring the interpersonal component of the mentoring relationship: The mentorship working alliance scale – mentee version

**DOI:** 10.1017/cts.2025.72

**Published:** 2025-04-16

**Authors:** Jenna Griebel Rogers, Angela Byars-Winston

**Affiliations:** 1 Center for the Improvement of Mentored Experiences in Research, Wisconsin Center for Educational Research, University of Wisconsin-Madison, Madison, WI, USA; 2 Institute for Diversity Science, Department of Medicine, University of Wisconsin-Madison, Madison, WI, USA

**Keywords:** Measurement, working alliance, mentorship, mentoring relationships, mentee career development

## Abstract

**Introduction:**

The interpersonal and relational dimensions of mentoring have been identified as critical components of effective mentorship. However, no scale currently exists to assess this specific aspect of the relationship. This study introduces a new instrument, the mentorship working alliance (MWA) – mentee version, and presents initial evidence supporting its validity in evaluating the interpersonal elements of mentoring relationships.

**Methods:**

Through a series of pilot tests and revisions, we developed a 12-item scale that assesses two dimensions of the MWA: relational quality (6 items), which captures how a mentee feels about the relationship, and relational effectiveness (6 items), which reflects the mentee’s perception of their mentor’s actions in facilitating or advancing the working relationship. To evaluate the scale’s construct validity and reliability, we conducted a confirmatory factor analysis (CFA) and internal consistency reliability analysis on a sample of 345 graduate students.

**Results:**

CFA provided evidence for the validity of the two-dimensional MWA scale, which assesses relational quality and relational effectiveness, with Cronbach’s alpha coefficients of 0.96 and 0.89, respectively. All parameter estimates for individual items were significant, with standardized factor loadings ranging from 0.66 to .83.

**Conclusions:**

The MWA scale – mentee version enables researchers to assess the interpersonal dimensions of mentoring relationships, offering valuable insights into the components of effective mentorship. By introducing this scale, we pave the way for further investigation into how mentorship interventions influence the MWA, thereby enhancing the overall quality of mentoring experiences. Additionally, we offer recommendations for future studies.

## Introduction

Effective mentorship plays a significant role in developing and diversifying the scientific workforce and is a critical determinant of mentees’ long-term persistence and academic success in research career pathways [1]. Central to this process is the mentoring relationship, which holds particular significance in supporting individuals from groups historically underrepresented in the sciences [1–3]. Research indicates that effective mentoring relationships positively influence mentees’ research-related learning experiences [4] and that mentees’ perceptions of these relationships impact their research self-efficacy, behavioral intentions, and persistence [5,6].

Mentoring relationships are enacted across many forms and for different career stages. Over the past few decades, the concept of mentoring has evolved beyond the traditional dyadic model, which assumes that a single mentor is sufficient to provide the wisdom and expertise needed to meet all of a mentee’s needs [7]. This expanded definition now includes alternative structures such as mentors and mentees in triads and mentoring networks as well as peer mentoring formed with same career stage colleagues [1]. In addition to diverse structures, mentorship encompasses a variety of behaviors associated with roles such as coaching, advising, role modeling, and sponsorship, all of which fulfill the psychosocial and career support functions of mentorship [1]. Concurrently, the scope of mentorship activities and behaviors has broadened, with increased emphasis on the psychosocial elements of the mentorship experience, which highlight the interpersonal and relational aspects of the mentoring [8,9].

Despite the well-documented importance of mentoring relationships, the specific elements that contribute to their effectiveness across diverse participants and contexts remain unclear. In 2019, the National Academies of Sciences, Engineering, and Medicine (NASEM) defined the mentoring relationship as a professional working alliance (WA). This conceptualization underscores the interpersonal, contextual, and relational components of close, dyadic relationships. It also emphasizes the importance of understanding how these components influence mentorship experiences and contribute to mentees’ persistence.

Recent studies have explored the various components of research mentoring relationships and the factors that contribute to their effectiveness. Pfund *et al*. [10] identified five key domains of effective research mentoring: research development, psychosocial and career support, cultural responsiveness, sponsorship, and interpersonal dynamics. Similarly, other studies have highlighted career development, psychosocial support, and role modeling as essential dimensions of mentoring [11]. While the specific elements of effective mentoring may vary, the interpersonal aspect consistently emerges as a critical factor. For instance, research indicates that a strong connection and effective communication between mentors and mentees are vital for successful mentoring relationships [12,13]. Additionally, studies examining mentor–mentee concordance have found that mentorship quality is less influenced by shared gender, race, or ethnicity and more by shared values, beliefs, and attitudes [14]. These findings reinforce the significance of the relational and interpersonal components of mentoring. Despite this, no dedicated scale currently exists to assess the interpersonal aspects of mentoring relationships. Previous studies have relied on proxy measures, such as relationship quality or overall mentoring effectiveness [4]. While these measures capture the role of the mentoring relationship within broader models, they fail to isolate the interpersonal components, which could influence other aspects of the relationship and ultimately its effectiveness.

One construct that has decades of empirical support for its facilitative role in developmental relationships is the WA introduced by Bordin [15] based on psychotherapy research. He defined the WA as an interpersonally negotiated exchange between a person who is seeking to change and the one who offers to facilitate that change. The WA construct has been applied to other developmental and professional training dyadic relationships like teacher-student, physician-patient, and supervisor–supervisee [16,17]. Schlosser and Gelso [18] extended the WA concept to assess the relational aspects of the doctoral advisor–advisee relationship. Based on their validated measure called the Advisory Working Alliance Inventory, they found strong associations of scale scores with advisees’ relationship satisfaction with their advisors and importantly with advisee outcomes including career interests formation and research self-efficacy beliefs [19]. Byars-Winston, Rogers, Branchaw and Pfund [20] found utility in applying the WA construct to the unique relational aspects of research mentoring relationships in the life and physical sciences and proposed the mentorship working alliance framework. The current study builds on this body of research to advance a measure of the mentorship working alliance.

While previous studies have examined effective mentoring strategies, few have focused on the relational context in which mentoring occurs. This study addresses this gap by introducing the mentorship working alliance (MWA) scale, designed to assess the interpersonal aspects of mentoring relationships. Although both mentors and mentees play crucial roles in research mentoring, this paper centers on the validation of the MWA scale from the mentee’s perspective. Ongoing research is validating the MWA scale for mentors, with findings to be reported in a future publication. We anticipate that the MWA Scale – Mentee Version will advance the science of mentorship research by providing a tool to evaluate mentees’ experiences of the interpersonal dimensions of mentoring within their research contexts. These insights will help inform intervention models aimed at optimizing mentorship experiences.

## Methods

### Development of the Scale

**Figure 1.**
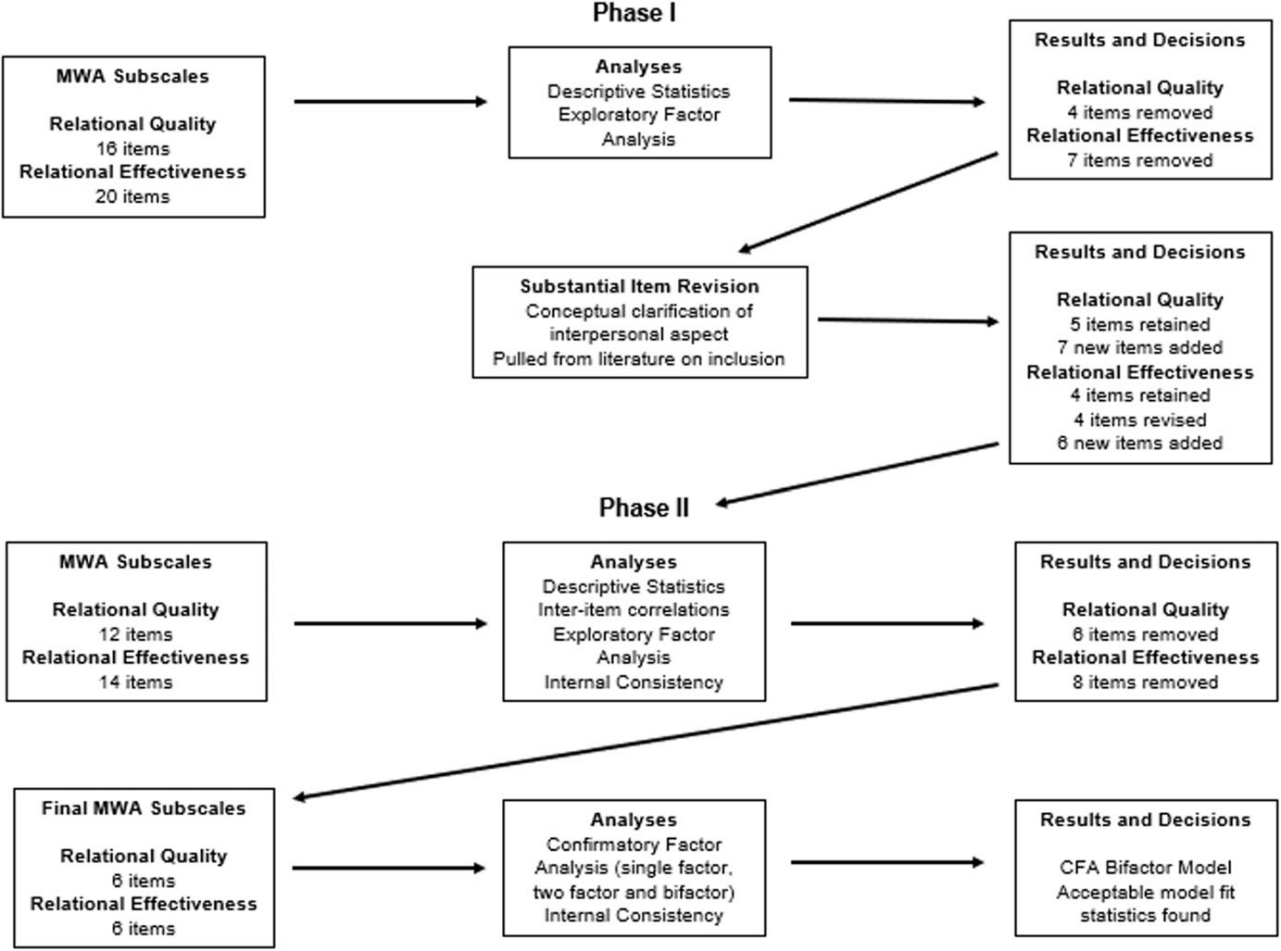
Validation phases for mentorship working alliance scale- mentee version. CFA = confirmatory factor analysis, MWA = Mentorship Working Alliance.

This study followed the four stages of scale development as outlined by Netemeyer et al. (2003) [21]. First, we defined the construct through an extensive theoretical and literature review. Second, we generated measurement items. Third, we refined the scale through preliminary testing, and finally, we conducted additional testing to finalize the scale. The development process comprised two main phases: Phase One included item generation, pilot testing, and revision (corresponding to stages one through three), while Phase Two focused on validity testing (aligned with stage four). Detailed descriptions of each phase are provided in this section. The study received approval from the institutional review board (protocols #2016-1517; #2019-1240). A detailed overview of the scale development process can be found in Fig. 1.

## Methods: phase I

### Participants

Phase I of the scale development process utilized data collected from fellows in the Gilliam Program at the Howard Hughes Medical Institute (HHMI). Gilliam Fellows are distinguished predoctoral trainees from groups historically underrepresented in the biomedical sciences. HHMI partnered with the Center for the Improvement of Mentored Experiences in Research to evaluate its Mentorship Skills Development Course. That partnership includes an annual electronic survey administered annually to both mentors and fellows (mentees) in the Gilliam Program which allowed data to be collected on several program and mentorship outcomes. The authors of this study helped lead the Gilliam annual surveys. For this study, they incorporated additional questions into the annual Gilliam Fellow surveys conducted in 2021, 2022, and 2023. This population provided a national sample of doctoral trainees from culturally diverse backgrounds and their mentors in the sciences representing dozens of research-intensive universities in the USA. Details regarding the sample size and composition are provided in the sections below.

### Item Generation

The generation of item content followed a rigorous, multi-step process. In Spring 2022, the research team conducted a comprehensive review of general mentorship scales (see Hernandez [22] for a detailed overview) and those specifically designed to evaluate mentorship quality and effectiveness. These included the Mentorship Effectiveness Scale [4]; the Mentoring Competency Assessment [23]; the Mentor Satisfaction Scale [24]; and another Mentorship Effectiveness Scale [25]. Additionally, we reviewed the adapted Working Alliance Inventory for Advisees/Advisors, developed by Schlosser and Gelso [18,19]. These instruments contain items addressing relational, psychosocial, instrumental, and networking functions of mentorship. From this analysis, we identified items focusing on relational aspects and mentorship behaviors as particularly relevant for inclusion in the MWA scale.

To ensure that the dominant themes in effective mentoring relationships from a mentee’s perspective were not overlooked, we also reviewed open-ended responses from mentees. To capture mentees’ perspectives on the qualities that define a strong mentoring relationship, we analyzed qualitative data from the Fall 2021 annual surveys of Gilliam Fellows (*N* = 50). Participants were asked to describe the characteristics of a “good” or “high-quality” mentoring relationship and to identify 2–3 elements necessary for their satisfaction within such relationships. The research team conducted thematic coding of the qualitative responses, revealing several recurring themes: openness, respect, support, compassion, and trust. These themes were then compared to the working list of items to ensure that all domains were represented in our initial items.

Based on the information outlined above, we developed initial items for the MWA scale. These items were reviewed by the study team for clarity, relevance, and phrasing, resulting in a 36-item scale designed to assess two dimensions of the Mentorship Working Alliance: relational quality (16 items) and relational effectiveness (20 items). The *relational quality* dimension evaluates the bond or rapport between the mentee and mentor. This subscale prompts mentees to reflect on their current mentoring relationship and rate their agreement, using a 5-point Likert scale, with statements that assess the strength of their connection with their mentor. For example, mentees are asked how much they agree or disagree with the statements “I feel supported by my mentor,” “I see my mentor and I as a part of the same team” and “I feel as though my mentor believes in me.” The *relational effectiveness* dimension focuses on mentor behaviors that enhance the interpersonal aspects of the relationship and influence its overall quality. This subscale asks mentees to report how frequently their mentor demonstrates specific supportive behaviors, with response options ranging from 1 (never) to 5 (all the time). For example, mentees are asked how frequently their mentor “initiated conversation about their well-being and mental health,” “validated their competence and potential for success” and “showed curiosity about who they are and what matters to them.”

### Analysis

Pilot survey items were integrated into the annual evaluation survey for the Gilliam Fellows Program in Fall 2022 and 2023 (*N* = 65). Among participants, 49% identified as female, 31% as male, and 11% did not report their gender. The sample included a diverse representation of racial and ethnic groups: 47% identified as Hispanic/Latinx, 12% as American Indian or Alaskan Native, 8% as Asian, 28% as Black or African American, 30% as white, and 10% as other.

We first examined item-level data to identify and remove problematic items based on mean, standard deviation, and distribution patterns. Each item’s mean, standard deviation, and distribution were analyzed to assess normality, with problematic items flagged for exclusion. Additionally, we reviewed response categories and item correlations within each subscale, removing items that exhibited poor performance. Preliminary exploratory factor analyses were conducted separately for the two subscales using principal axis factoring with varimax rotation. Principal axis factoring (PAF) was chosen because it is better suited for data that may not adhere to a normal distribution and emphasizes the underlying latent constructs [26]. Varimax rotation was selected because we assumed that the two subscales were unidimensional and that factors should be orthogonal [27]. Following the Kaiser criterion, factors with eigenvalues greater than 1 were retained [28]. Scree plots were also examined to visually confirm the number of factors, ensuring that retained factors aligned with the theoretical structure of the two subscales. Following Tabachnick and Fidell’s [29] suggestion of using more stringent cutoffs ranging from 0.32 (poor), 0.45 (fair), 0.55 (good), 0.63 (very good) or 0.71 (excellent), items with factor loadings below 0.45 or those that loaded onto multiple factors were flagged for review and removed to ensure the clarity and construct validity of the subscales. A detailed layout of all steps and decision points can be found in Fig. 1.

## Results: phase I

We began by analyzing the relational quality scale. Preliminary item review found no items with concern regarding distribution of scores. Exploratory factor analysis resulted in the immediate removal of four items from the relational quality subscale and seven items from the relational effectiveness subscale. Examination of scree plots (see Fig. 1 and 2 in supplemental materials) and eigenvalues revealed multiple factors for both relational quality and relational effectiveness. Items were removed from both subscales due to low factor loadings, high cross-loadings across multiple factors, or a failure to align with the intended conceptual definitions of relational quality and relational effectiveness. A closer examination revealed that, contrary to the initial assumption of uni-dimensionality, items loaded onto multiple factors. This issue was especially pronounced within the relational effectiveness subscale, where several items exhibited split loadings across multiple factors. Split loadings, where items loaded onto multiple factors, suggested that those items were tapping into different constructs rather than the intended, unidimensional aspect of relational effectiveness. This undermined the scale’s conceptual clarity and its ability to accurately measure the intended construct. These findings suggested to the research team that certain items required revision to improve the scale’s conceptual clarity.

**Figure 2.**
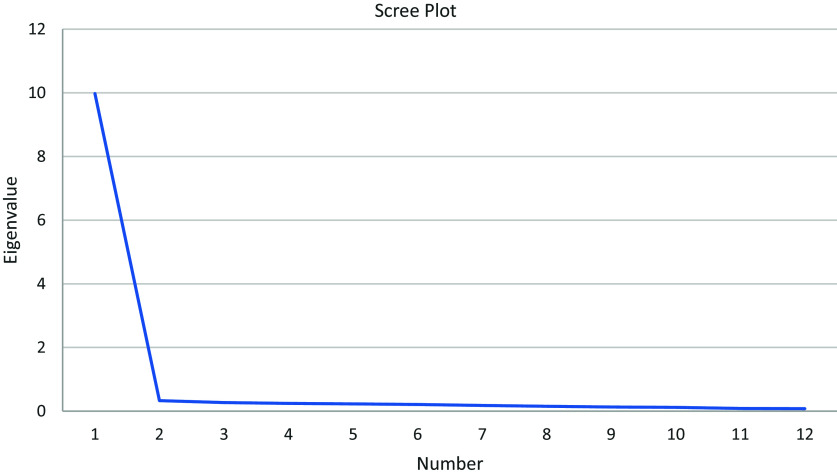
Scree plot of the eigenvalues of the factors of relational quality scale.

### Item revision

Based on the initial pilot testing of the MWA items, the research team identified the need for substantial item revisions. The team reaffirmed that the MWA scale was designed to measure the interpersonal and relational dimensions of the mentoring relationship, comprising two core constructs: relational quality (the mentee’s perceptions of the mentoring relationship, including bond and rapport) and relational effectiveness (the mentor’s behaviors that enhance relational quality). Crucially, the team clarified that relational effectiveness pertains specifically to behaviors that support the interpersonal aspects of the relationship. This distinction was essential, as many initial items within the relational effectiveness subscale addressed general mentoring behaviors rather than interpersonal-specific actions, which contributed to multiple-factor loadings in the analysis. While these general behaviors undoubtedly promote mentee success and enrich the overall mentoring experience, they do not directly enhance the interpersonal bond or strengthen the mentor-mentee relationship. To address this issue, we drew upon the literature on inclusion, which highlights relational patterns and practices that foster safe, supportive environments. Inclusive practices are characterized by interactions that make individuals feel valued, cared for, and a sense of belonging [30–33]. These principles guided the revision of items to better capture the relational effectiveness dimension within the context of mentoring [31].

With this conceptual clarification, the research team conducted a comprehensive item review, removing items that did not align with the interpersonal focus of the mentoring relationship. Drawing on research into inclusive environments and the behaviors that promote them, the team developed new items that reflected this perspective. After conducting the item revision process and ensuring alignment with the interpersonal focus of the mentoring relationship, the team developed a 14-item relational effectiveness scale and a 12-item relational quality scale.

## Methods: phase II

### Participants

In the second phase of this study, we surveyed graduate students from biomedical science departments within research training programs at 12 research-intensive universities in the United States. These sites were selected based on their prior involvement in an NIH-supported study on Culturally Aware Mentoring (see Eiring *et al*. [34] for a detailed methodology) led by one of the authors of this study. Like the Gilliam sample in Phase I, this sample provided a national sample of doctoral trainees in research-intensive universities. Each site was invited to distribute an electronic survey link to all graduate students in their programs. To encourage participation, respondents were offered a $10 gift card incentive. Of the 30 sites invited, 12 agreed to participate, yielding a total of 420 survey responses. After excluding incomplete surveys, the final sample comprised 345 participants. Detailed participant demographics are presented in Table 1.

**Table 1.** Characteristics of mentees in phase II

Characteristics	Mentees (N = 345)
**Gender, no. (%)**	
Female	218 (63.1)
Male	110 (31.9)
Other	7 (2.0)
Prefer not to report	10 (2.8)
Combined race/ethnicity^*^, no. (%)	
American Indian or Alaskan Native	5 (1.4)
Asian	94 (27.2)
Black or African American	24 (7.0)
Hispanic Or Latinx	35 (10.1)
White	192 (55.7)
Other	15 (4.3)
Prefer not to report	12 (3.5)
**Duration of mentoring relationship, no. (%)**	
Less than 1 year	78 (22.6)
1–2 years	80 (23.2)
2–3 years	60 (17.4)
3–4 years	57 (16.5)
Longer than 4 years	70 (20.3)

* Participants can select more than one option.

### Analysis

Exploratory factor analyses were conducted for the two subscales using principal axis factoring with varimax rotation. Following the Kaiser criterion, factors with eigenvalues greater than 1 were retained [28]. Scree plots were also examined to visually confirm the number of factors and ensure the structure of the two subscales. Descriptive statistics and inter-item correlations were calculated, including mean scores and standard deviations for both subscales. Internal consistency was assessed using Cronbach’s α coefficient, a widely accepted metric for evaluating the reliability of a scale [21]. Cronbach’s α values range from 0 to 1, with a value of 0.70 or higher typically indicating good internal consistency [21]. Items were flagged for potential removal if their exclusion improved the scale’s internal consistency (i.e., if their Cronbach’s α value increased when the item was removed) or if they had an item-total correlation below 0.3. To account for the potential multi-dimensionality of the scales McDonald’s Omega coefficient was also used to examine scale reliability, with values of 0.70 or higher indicating strong reliability [35].

Scale construct validity was evaluated through a series of confirmatory factor analysis (CFA) using maximum-likelihood estimation in EQS statistical software. Maximum-likelihood estimation was used for the CFAs because it is a robust method that performs well with normally distributed data and provides estimates of model fit that are reliable and interpretable. Model fit was assessed based on key indices, including the comparative fit index (CFI), Tucker–Lewis Index (TLI), standardized root-mean-square residual (SRMR), and root-mean-square error of approximation (RMSEA) [36–38]. According to established guidelines, an RMSEA value exceeding 0.10 reflects poor model fit, while an SRMR below 0.08 and CFI and TLI values above 0.95 signify good fit [36,39].

## Results: phase II

**Figure 3.**
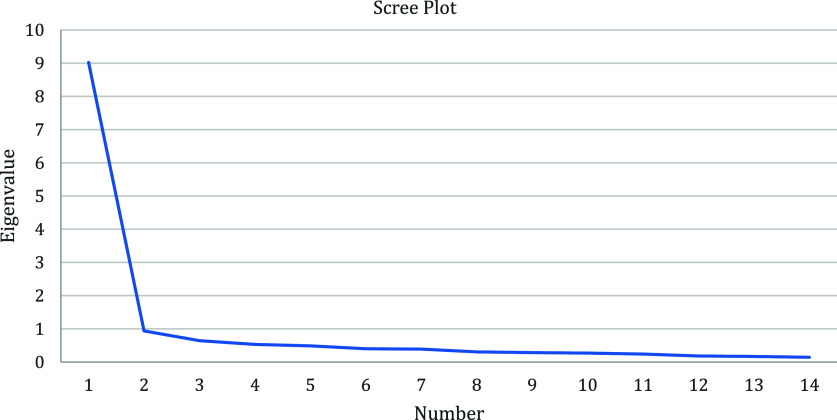
Scree plot of the eigenvalues of the factors of relational effectiveness scale.

Examination of scree plots (see Figs. 2 and 3) and eigenvalues revealed a single factor for both relational quality and relational effectiveness with high factor loadings for each of the items, aligning with our theorized subscales. An examination of descriptive statistics and inter-item correlations (Table 2) and alpha coefficients led to the removal of six items from the relational quality scale and eight items from the relational effectiveness scale. Of note is that the six remaining relational quality items had high inter-item correlations. After discussing this as a team, we choose to keep all six items as the high correlations made sense due to the narrow focus of this construct. Additionally, items on the relational quality subscale tended to skew with most mentees rating their relational quality strongly, leading to a high mean for relational quality. These items were examined in depth. Measures of skewness ranged between 0.5 and 1.4, and every response category was used within each item, reflecting that the item is sensitive enough to capture all ranges of responses. Thus, the skew was hypothesized to be a reflection of the sample (see discussion). The final alpha (omega) coefficients for the two subscales were 0.959 (0.960) and 0.893 (0.884), respectively, indicating strong internal consistency and reliability for both scales. Table 3 provides the factor loadings and internal consistency statistics for the MWA scale. A CFA using maximum-likelihood estimation in EQS demonstrated that a two-factor solution fit the data well. The fit indices were χ^2^ = 209, *p* < 0.001, RMSEA = 0.093, CFI = 0.958, TLI = 0.948, and SRMR = 0.043. All parameter estimates for each item were statistically significant, with standardized factor loadings ranging from 0.65 to 0.89. These high loadings indicate that the items strongly align with their respective factors, supporting the validity of the two subscales. Table 4 presents the CFA results alongside descriptive statistics.

**Table 2.** Descriptive statistics and inter-item correlations

*Item*	*M*	*(SD)*	RQ1	RQ2	RQ3	RQ4	RQ5	RQ6	RE1	RE2	RE3	RE4	RE5	RE6
RQ1: I feel supported by my mentor	4.26	1.11	–	.800^**^	.819^**^	.782^**^	.804^**^	.789^**^	.526^**^	.667^**^	.527^**^	.563^**^	.493^**^	.648^**^
RQ2: I feel as though my mentor and I get along	4.3	1.02		–	.797^**^	.779^**^	.791^**^	.816^**^	.481^**^	.666^**^	.489^**^	.503^**^	.519^**^	.620^**^
RQ3: I feel valued by my mentor	4.16	1.20			–	.810^**^	.862^**^	.809^**^	.545^**^	.713^**^	.516^**^	.560^**^	.477^**^	.662^**^
RQ4: I feel understood by my mentor	3.77	1.28				–	.783^**^	.781^**^	.571^**^	.696^**^	.590^**^	.574^**^	.544^**^	.655^**^
RQ5: I feel as though my mentor believes in me	4.25	1.11					–	.799^**^	.514^**^	.683^**^	.477^**^	.565^**^	.459^**^	.633^**^
RQ6: I see my mentor and I as a part of the same team	4.27	1.12						–	.501^**^	.661^**^	.503^**^	.522^**^	.506^**^	.628^**^
RE1: Initiated conversations with you about your well-being and mental health	2.95	1.31							–	.617^**^	.750^**^	.516^**^	.479^**^	.615^**^
RE2: Validated your competence and potential for success	3.83	1.12								–	.647^**^	.590^**^	.512^**^	.657^**^
RE3: Encouraged you to talk openly about your anxieties and fears	3.09	1.39									–	.554^**^	.514^**^	.598^**^
RE4: Shared some of their own past successes and challenges in their career development with you	3.73	1.21										–	.558^**^	.543^**^
RE5: Shared a social identity that they hold outside of being a scientist with you	3.27	1.31											–	.640^**^
RE6: Shown curiosity about who you are and what matters to you	3.57	1.25												–

** *Significant at P < 0.001*.

**Table 3.** Factor loadings and cronbach’s alpha scores for the 12 item mentorship working alliance scale

No.	Item	Factor loading	Alpha	Omega
**Relational quality subscale^a^**			0.960	0.960
RQ1	I feel supported by my mentor.	0.795		
RQ2	I feel as though my mentor and I get along.	0.773		
RQ3	I feel valued by my mentor.	0.817		
RQ4	I feel understood by my mentor.	0.825		
RQ5	I feel as though my mentor believes in me.	0.787		
RQ6	I see my mentor and I as a part of the same team.	0.776		
**Relational effectiveness subscale^b^**			0.893	0.893
RE1	Initiated conversations with you about your well-being and mental health.	0.676		
RE2	Validated your competence and potential for success.	0.821		
RE3	Encouraged you to talk openly about your anxieties and fears.	0.665		
RE4	Shared some of their own past successes and challenges in their career development with you.	0.693		
RE5	Shared a social identity that they hold outside of being a scientist with you.	0.671		
RE6	Shown curiosity about who you are and what matters to you.	0.811		

a For Relational Quality items, mentees were asked: “We would like for you to think about your current mentoring relationship. In the context of this working relationship, how much do you agree or disagree with the below statements;” responses could range from 1 (strongly disagree) to 5 (strongly agree) ^b^ For Relational Effectiveness items, mentees were asked: “Within your current mentoring relationship, how often has your mentor;” responses could range from 1 (never) to 5 (all of the time).

**Table 4.** Results of phase II confirmatory factor analyses

		Mean^*^	SD	RMSEA	SRMR	CFI	TLI
Two Factor Model	Relational Quality	4.16	1.04	0.093	0.043	0.958	0.948
	Relational Effectiveness	3.41	1.02				
Bifactor Model	Relational Effectiveness	4.16	1.04	0.071	0.025	0.981	0.970
	Relational Quality	3.41	1.02				

* Range: 1–5.

Given the significant and high correlation between the two subscales (r = 0.765, *p* < 0.001), an additional CFA was conducted to evaluate whether the items might represent a single underlying dimension, potentially leading to artificial factor loadings. In this model, all items were loaded onto a single factor. The results demonstrated poor model fit, reinforcing the decision to retain the originally hypothesized two-factor structure.

**Figure 4.**
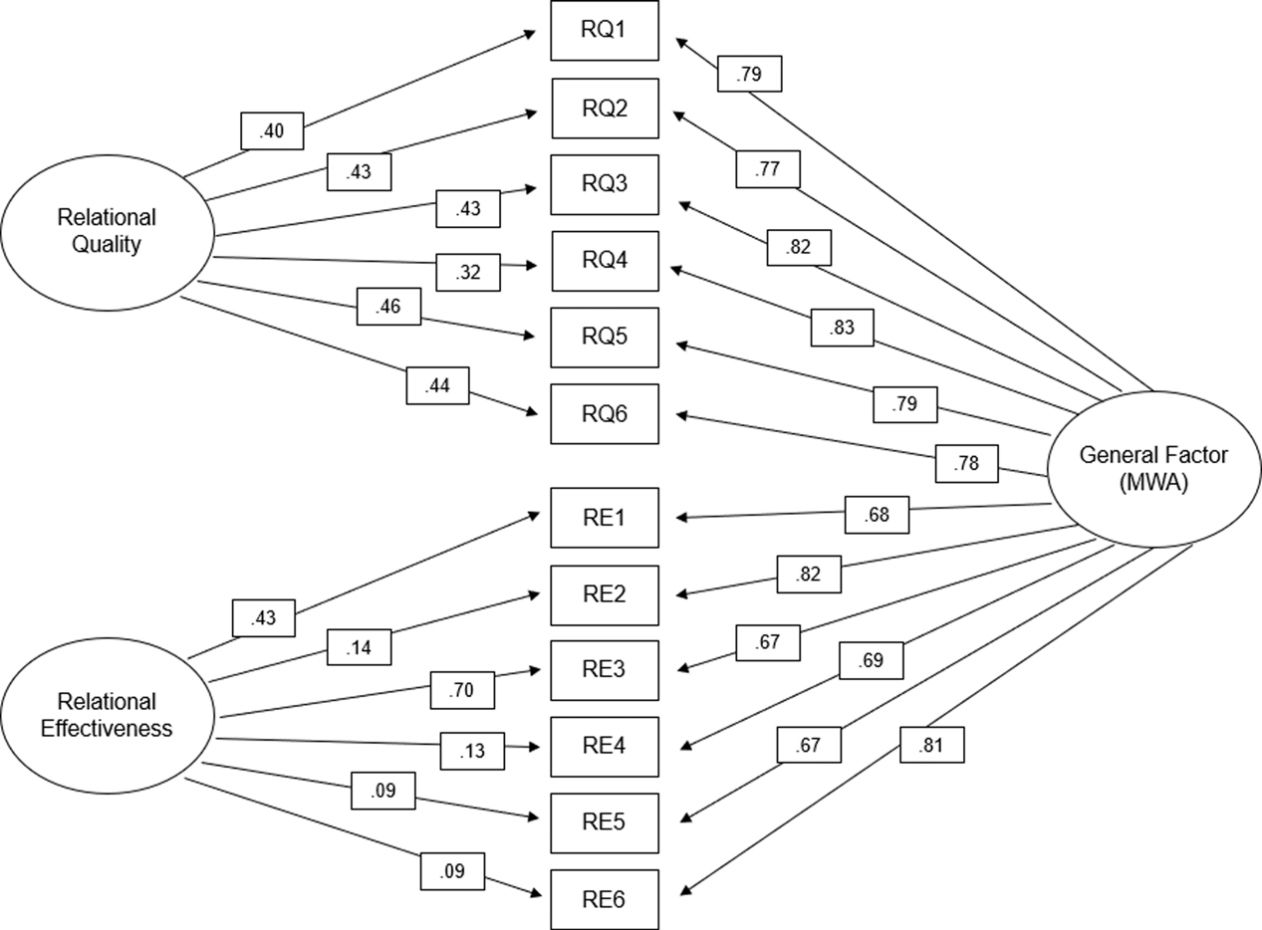
Bifactor confirmatory factor analysis Model. *All loadings were significant at the .05 level; Standardized solution. MWA = Mentorship Working Alliance.

A bifactor CFA model was next run to account for the high correlation between subscales. A bifactor CFA model can be used to analyze complex constructs with multiple sub-dimensions. A bifactor model can model a general, overarching factor as well as more specific sub-factors [40]. In this model, the general factor is the mentorship working alliance with the relational quality and relational effectiveness as sub-dimensions or sub-factors. The model and factor loadings are shown in Fig. 4. Fit indices were χ^2^ = 113, *p* < 0.001, RMSEA = 0.071, CFI = 0.981, TLI = 0.970, and SRMR = 0.025, (Table 3) demonstrating a strong fit. All parameter estimates were statistically significant, with standardized factor loadings ranging from 0.67 to .83. Factor loadings on the general factor (MWA) indicate the strength of the relationship between the observed variables and the overall construct while the loadings on the relational quality and relational effectiveness factors demonstrate how much each item contributes to the sub-dimension (while still being a part of the larger construct). The results show the bifactor model to be the best fit, and thus our final model.

## Discussion

The mentoring relationship is a critical component of the mentorship experience [4–6]. However, the specific factors that contribute to its effectiveness remain unclear. This study introduced and validated the MWA scale–mentee version, designed to assess the interpersonal component of mentoring relationships from the mentee’s perspective. Following a series of pilot tests and revisions, a 12-item scale was developed to evaluate two dimensions of the mentorship working alliance: relational quality (6 items), which captures how mentees feel about the relationship, and relational effectiveness (6 items), which reflects mentees’ perceptions of their mentor’s behaviors that facilitate or enhance the working relationship. The validated scale demonstrated strong potential to be a reliable tool for measuring the interpersonal and relational aspects of mentoring. Findings indicate that while relational quality and relational effectiveness are conceptually distinct, they are also closely related, as evidenced by the high correlation between the subscales. The bifactor model we found in our results provided evidence in support of our articulation of the mentorship working alliance construct. That is, the interpersonal aspect of the mentoring relationship encompasses not only the quality of the relationship, defined by the bond between mentor and mentee but also the behaviors exhibited by the mentor in service to the working relationship (relational effectiveness). This distinction is important, as previous scales have not simultaneously accounted for both dimensions when measuring the mentoring relationship in research studies, nor did a validated measure exist to assess these dimensions. This MWA scale assessing the mentees’ perspective will provide a more specified understanding of the interpersonal dynamics within mentorship.

There are distinctive factors concerning the samples we used in both stages of the scale development. In Phase I, the mentees were part of an intensive mentored research program specifically designed at the time for distinguished doctoral trainees from groups underrepresented in the sciences based on race, ethnicity, class, or ability status. The mentors in this program had completed a yearlong mentorship skills development curriculum that included elements of the evidence-based *Entering Mentoring* and *Culturally Aware Mentor* (CAM) trainings [3]. The mentees in Phase II were recruited based on their program’s previous participation in an efficacy study investigating the CAM intervention [28]. Consequently, a significant number of our study’s mentee participants had mentors who had received robust mentor training. Research studies indicate that less than 25% of research mentors have participated in a structured mentor training [41,42]. Thus, this sample may not represent a typical mentoring experience, which is more likely to be delivered by mentors who have less intensive mentor training or no training at all.

That the mentors of the current mentee sample had mentorship education may have contributed to the high mean scores on the relational quality subscale. Mentors who have had mentorship education reported enhanced skills in fostering effective mentoring relationships [41], which presupposes positive interpersonal interactions. Subsequent studies involving a broader cohort of mentees with untrained mentors could assess the variability in responses to the relational quality subscale items to confirm the absence of a ceiling effect.

The MWA Scale – mentee version provides researchers with a tool to assess the interpersonal dimensions of mentoring relationships, offering valuable insights into the elements that contribute to effective mentorship. Dolan *et al*. [14,32–34] explored how undergraduate and graduate students in the life sciences are both supported and hindered by the interpersonal dynamics of research mentoring relationships. Their findings indicate that less supportive or negative mentoring relationships are associated with reduced scientific integration and a diminished valuation of research. The MWA–mentee scale allows for a more targeted analysis of which aspect of the interpersonal relationship – the relational quality or the effectiveness of mentoring behaviors – is functioning well or needs improvement. Moreover, the scale offers potential applications as both a diagnostic self-assessment tool for evaluating the mentorship working alliance (MWA) and as a guide for interventions aimed at improving compromised mentoring relationships. Program training directors could administer the measure to their trainees and use the results to guide training interventions for their mentors that target the salient relational factors (effectiveness, quality) identified in the results. Such insights could pinpoint areas where new mentor trainings interventions need to be developed to further strengthen the mentorship working alliance.

### Future directions and recommendations

This study introduces the MWA scale, providing initial evidence of its validity in assessing the interpersonal dimension of mentoring relationships. While the findings are promising, further validation studies are needed, particularly those examining the scale’s predictive validity. Future research should explore how the MWA influences mentee academic and career development and persistence. The scale was developed and validated using a sample of graduate students. Although prior research indicates that mentees’ needs and expectations evolve throughout their academic and professional trajectories [46]. The interpersonal aspect of the mentoring relationship nonetheless remains pivotal. We hypothesize that the MWA may have a greater impact on early-career stage mentees, such as undergraduates and graduate students, who require more guidance than later-career mentees, such as postdoctoral scholars and early-career faculty, who have achieved comparatively greater career progress and autonomy. Despite the varying influence of the MWA across career stages, we anticipate that the interpersonal component will continue to play a significant role. Even mid-career professionals benefit from relational support through mentorship tailored to their career development needs like pursuing leadership roles and building their visibility [47]. Future studies should validate the MWA scale across career stages and investigate its impact on mentee outcomes.

This study focused on the mentee’s perspective of the MWA; however, the working alliance inherently involves both the mentee and the mentor. Thus, assessing the mentor’s perspective on the MWA is equally important. Future research should validate the MWA for mentors and investigate how their perceptions influence the overall mentorship experience.

The development of validated measures that assess multiple facets of research mentorship in the biomedical sciences is critical for advancing the science of effective mentorship. These measures enable the establishment of common standards across programs, facilitating the identification and analysis of factors that contribute to successful mentoring experiences across career stages and disciplines, including translational science. Training programs, such as Clinical and Translational Science Award initiatives, continue to prioritize the enhancement of mentorship training, practices, and behaviors. Validated instruments will be essential for assessing the impact of these efforts and demonstrating their effectiveness in fostering high-quality mentorship.

## Supporting information

Rogers and Byars-Winston supplementary material Rogers and Byars-Winston supplementary material

## Supplementary material

The supplementary material for this article can be found at https://doi.org/10.1017/cts.2025.72.
