# Supplementary material for: Measuring the interpersonal component of the mentoring relationship: The mentorship working alliance scale – mentee version
Source: J Clin Transl Sci. 2025 Apr 16;9(1):e99. doi: 10.1017/cts.2025.72 (PMC12089860; doi:10.1017/cts.2025.72)
Supplement: Rogers and Byars-Winston supplementary material [file S205986612500072Xsup001.docx]

**Supplementary Materials**

**Figure 1. Scree plot of the eigenvalues of the factors of Relational Quality Pilot Scale**

**Figure 2. Scree plot of the eigenvalues of the factors of Relational Quality Effectiveness Pilot Scale**
